# Supplementary material for: Resolving the full spectrum of human genome variation using Linked-Reads
Source: Genome Res. 2019 Apr;29(4):635–45. doi: 10.1101/gr.234443.118 (PMC6442396; doi:10.1101/gr.234443.118)
Supplement: Supplemental Material [file supp_gr.234443.118_Supplemental_longranger-2.2.zip › longranger-2.2.tar.bz2!/lr2.2-public/lib/stan/cmdstan/stan/doc/api/html/check__nonnegative_8hpp_source.html]

Stan: src/stan/math/error\_handling/check\_nonnegative.hpp Source File


|  |  |
| --- | --- |
|  | Stan  2.5.0  probability, sampling & optimization |


- Main Page
- Related Pages
- Namespaces
- Classes
- Files

- File List
- File Members

All Classes Namespaces Files Functions Variables Typedefs Enumerations Enumerator Friends Macros Pages

- src
- stan
- math
- error\_handling

check\_nonnegative.hpp

Go to the documentation of this file.

1 #ifndef STAN\_\_MATH\_\_ERROR\_HANDLING\_CHECK\_NONNEGATIVE\_HPP

2 #define STAN\_\_MATH\_\_ERROR\_HANDLING\_CHECK\_NONNEGATIVE\_HPP

3

4 #include <stan/math/error\_handling/dom\_err.hpp>

5 #include <stan/math/error\_handling/dom\_err\_vec.hpp>

6 #include <boost/type\_traits/is\_unsigned.hpp>

7 #include <stan/meta/traits.hpp>

8 #include <stan/math/meta/value\_type.hpp>

9

10 namespace stan {

11

12  namespace math {

13

14  namespace {

15  template <typename T\_y, typename T\_result, bool is\_vec>

16  struct nonnegative {

17  static bool check(const char\* function,

18  const T\_y& y,

19  const char\* name,

20  T\_result\* result) {

21  // have to use not is\_unsigned. is\_signed will be false

22  // floating point types that have no unsigned versions.

23  if (!boost::is\_unsigned<T\_y>::value && !(y >= 0))

24  return dom\_err(function,y,name,

25  " is %1%, but must be >= 0!","",

26  result);

27  return true;

28  }

29  };

30

31  template <typename T\_y, typename T\_result>

32  struct nonnegative<T\_y, T\_result, true> {

33  static bool check(const char\* function,

34  const T\_y& y,

35  const char\* name,

36  T\_result\* result) {

37  using stan::length;

38  for (size\_t n = 0; n < length(y); n++) {

39  if (!boost::is\_unsigned<typename value\_type<T\_y>::type>::value

40  && !(stan::get(y,n) >= 0))

41  return dom\_err\_vec(n,function,y,name,

42  " is %1%, but must be >= 0!","",

43  result);

44  }

45  return true;

46  }

47  };

48  }

49

50  // throws if any element in y is nan

51  template <typename T\_y, typename T\_result>

52  inline bool check\_nonnegative(const char\* function,

53  const T\_y& y,

54  const char\* name,

55  T\_result\* result) {

56  return nonnegative<T\_y,T\_result,is\_vector\_like<T\_y>::value>

57  ::check(function, y, name, result);

58  }

59  }

60 }

61 #endif

dom\_err.hpp

stan::math::dom\_err

bool dom\_err(const char \*function, const T &y, const char \*name, const char \*error\_msg, const T\_msg error\_msg2, T\_result \*result)

**Definition:** dom\_err.hpp:36

stan::length

size\_t length(const T &)

**Definition:** traits.hpp:159

stan::math::dom\_err\_vec

bool dom\_err\_vec(const size\_t i, const char \*function, const T &y, const char \*name, const char \*error\_msg, const T\_msg error\_msg2, T\_result \*result)

**Definition:** dom\_err\_vec.hpp:33

traits.hpp

stan::math::check\_nonnegative

bool check\_nonnegative(const char \*function, const T\_y &y, const char \*name, T\_result \*result)

**Definition:** check\_nonnegative.hpp:52

value\_type.hpp

dom\_err\_vec.hpp

stan::get

T get(const T &x, size\_t n)

**Definition:** traits.hpp:143


---

[ Stan Home Page ]

*© 2011–2014,
Stan Development Team.*
